# Special Issue “Biomaterials and Bioprinting”

**DOI:** 10.3390/molecules21091231

**Published:** 2016-09-14

**Authors:** Chee Kai Chua, Wai Yee Yeong, Jia An

**Affiliations:** Singapore Centre for 3D Printing, School of Mechanical and Aerospace Engineering, Nanyang Technological University, Singapore 639798, Singapore; WYYeong@ntu.edu.sg (W.Y.Y.); AnJia@ntu.edu.sg (J.A.)

**Keywords:** biomaterial, nanobiomaterial, hydrogel, tissue spheroids, tissue model, bioprinting, biofabrication, biomanufacturing, 3D printing, additive manufacturing, rapid prototyping

## Abstract

The emergence of bioprinting in recent years represents a marvellous advancement in 3D printing technology. It expands the range of 3D printable materials from the world of non-living materials into the world of living materials. Biomaterials play an important role in this paradigm shift. This Special Issue focuses on biomaterials and bioprinting and contains eight articles covering a number of recent topics in this emerging area.

Bioprinting represents a new frontier of 3D printing technology. The process involves the incorporation of living cells or other biological elements with biomaterials for robotic and automated tissue manufacturing. Driven by innovations in 3D printable biomaterials and breakthroughs in 3D bioprinting technology, the market size of bioprinting is forecasted to reach US$615 million by 2024 and US$10 billion by 2030. The huge potential of this emerging field motivated this Special Issue.

Bioprinting never involves a single technology. The bioprinting family consists of at least extrusion, inkjet and laser assisted material transfer techniques. Moreover, computer-aided design, cells, biomaterials, bioprinter, bioreactor and preclinical trials, all are necessary components in an integrated bioprinting process chain. Therefore, bioprinting is a comprehensive multidisciplinary research field that involves engineering, biology, material sciences and many more beyond. This leads to a very wide scope in bioprinting research. In this Special Issue, we choose to focus on biomaterials and bioprinting, because this topic represents the fastest growth in this area.

This Special Issue includes four review articles and four original research articles, covering the most recent topics in biomaterials and bioprinting research. In review articles, Lei and Wang presents various bioprinting techniques and the use of stem cells and biodegradable polymers in bioprinting [1]. They argue that bioprinting, serving as an integration platform for combined, single-step fabrication of cell-scaffold constructs, brings huge opportunities for tissue repair and organ transplantation. Panwar and Tan review current status and challenges of bioink development for extrusion-based 3D bioprinting [2]. They point out that bioinks are cell-specific and hence many challenges ahead, but the foremost is to solve the printability issue. Zhou introduces a freshly new approach to bioprinting by exploring the role of ultrasound [3]. He demonstrates that ultrasound could help achieve uniform cell distribution and guide stem cell differentiation at various stages of bioprinting process. Irvine and Venkatraman focus their review particularly on the control of differentiation of bioprinted stem cells [4]. They propose that bioprinted stem cell differentiation could be a new form of 4D bioprinting.

In research articles, Palomeras et al. investigate the role of 3D printing for building 3D cancer models. They discover that the use of 3D printed polycaprolactone (PCL) scaffolds could significantly improve the mammosphere forming index of cancer stem cells [5]. Suntornnond et al. are interested in new fabrication methods. They report that a thin PCL layer, which may potentially be used for layer by layer biofabrication, could be simply produced by heating a layer of PCL particles [6]. Similarly, Vaezi et al. present a new biofabrication method. They demonstrate the extrusion of polyetheretherketone (PEEK) and hydroxyapatite (HA) composites in a unique configuration, in which 3D HA network was embedded in the PEEK matrix [7]. Floroian et al. develop a coating method for 3D printed metallic implants [8]. They show that printing of novel bioactive glass-polymer-antibiotic composites into uniform thin films onto 316 L stainless steel substrates could shield metal ion release, improve biocompatibility and resist biofilm formation.

In summary, bioprinting is emerging and the research area of biomaterials for bioprinting is rapidly expanding with enormous scope for advancement and applications. We would like to express our sincere gratitude to all of the contributing authors for their selfless devotion of time and efforts to this Special Issue.
